# Sperm recovery from urine in men with retrograde ejaculation

**DOI:** 10.1515/almed-2024-0109

**Published:** 2024-08-22

**Authors:** Ernesto Veiga Álvarez, Nuria Zopeque García, Javier M. Gutiérrez Romero, Pilar Reimundo Díaz-Fierros, María D. Lozano Arana, Tamara Rodríguez Pérez, Javier Sánchez Álvarez, Guadalupe Bueno Rodríguez, Vanesa Castañón Bernardo, María J. Moyano Gallego

**Affiliations:** Working Group on Andrology and Assisted Reproduction Technologies, Spanish Society of Laboratory Medicine (SEQC^ML^),, Barcelona, Spain; Assisted Human Reproduction Unit, Central Laboratory, University Clinical Hospital of Santiago de Compostela, Santiago de Compostela, Spain; Laboratory of Assisted Reproduction, Service of Biochemistry. Alcorcón Foundation University Hospital, Calle Budapest, 1, 28922 Alcorcón, Madrid, Spain; Laboratory of Assisted Reproduction, Service of Biochemistry, Puerta del Mar University Hospital, Cadiz, Spain; Laboratory of Assisted Reproduction and Andrology, Service of Clinical Biochemistry, Vall d’Hebron Clinical Laboratories, Vall d’Hebron University Hospital, Pg. de la Vall d’Hebron, 119, 08035, Barcelona, Spain; Laboratory of Assisted Reproduction, UGC of Maternal Fetal Medicine, Genetics and Reproduction, Women’s Hospital, Virgen del Rocío University Hospital, Avda. Manuel Siurot s/n, 41013, Sevilla, Spain; Laboratory of Andrology and Assisted Reproduction, Service of Biochemistry, Women’s Hospital, La Paz University Hospital, Paseo de la Castellana 261, 28046, Madrid, Spain; Laboratory of Assisted Reproduction and Andrology, Service of Clinical Biochemistry, Vall d’Hebron Clinical Laboratories, Vall d’Hebron University Hospital, Pg. de la Vall d’Hebron, 119, 08035, Barcelona, Spain; Laboratory of Assisted Reproduction and Andrology. Service of Biochemistry. Virgen de Valme University Hospital, Avda. Bellavista s/n, 41014, Sevilla, Spain; Vanesa Castañón Bernardo, Laboratory of Assisted Reproduction. Central University Hospital of Asturias. Avda. Roma s/n. 33011 Oviedo, Spain; Laboratory of Assisted Reproduction; UCG Clinical Analysis. Reina Sofía University Hospital. Avenida Menéndez Pidal s/n 14004, Córdoba, Spain

**Keywords:** aspermia, azoospermia, ejaculation, male infertility, semen, sexual dysfunction

## Abstract

**Introduction:**

Retrograde ejaculation (RE) consists of the reflux backwards, towards the bladder, of the ejaculate, during the emission phase of ejaculation, causing a total or partial absence of sperm emission, with the consequent diversion of semen into the bladder during the emission phase of ejaculation. Evaluating the ejaculate may not be sufficient for identifying RE in some patients. Hence, the management of infertility may involve the use of invasive methods such as epididymal fluid retrieval or testicular biopsy.

**Content:**

This paper defines RE and methods for its diagnosis. A description is also provided of the techniques used for the detection of sperm in post-ejaculatory urine (PEU), the preparation and retrieval of sperm from urine and their subsequent use in assisted reproductive techniques.

**Summary:**

The diagnosis of RE is based on the detection of spermatozoa in PEU in patients with aspermia or oligozoospermia and low or normal seminal volume. Although the presence of sperm in PEU could be sufficient for a diagnosis of RE, there is a lack of consensus regarding the diagnostic criteria for PEU, and the literature available is very limited. A correct diagnosis of RE allows the use of PEU for recovering sperm and its subsequent use in assisted human reproduction techniques, thus avoiding invasive techniques.

**Outlook:**

A significant number of patients with RE may remain undiagnosed. Therefore, it is essential to conduct an RE study in patients with suspicion, through the analysis of PEU, and to properly interpret the results for accurate diagnosis.

## Introduction

During ejaculation, the internal urethral sphincter (IUS) must remain closed to prevent retrograde ejaculation (RE), which is the partial or total backward flow of semen into the bladder [1].

The most common causes of RE include surgeries that cause bladder neck incompetence, the use of drugs or radiotherapy for treating cancer in the pelvic region, multiple sclerosis, dissection of retroperitoneal lymph nodes, bone marrow lesions, secondary neuropathy in patients with diabetes mellitus (DM), or idiopathic causes [2], [3], [4], [5], [6], [7], [8], [9], [10], [11], [12], [13], [14], [15], [16], [17].

Typical RE is characterized by aspermia followed by urination with cloudy urine, although it can also present wih hypospermia (ejaculate volume <1.4 mL) and oligozoospermia, and occasionally even normal seminal volume can be observed [4, 18], [19], [20]. On another note, the presence of hypospermia and azoospermia with palpable vas deferens may be caused by ejaculatory duct obstruction or, in some cases, by ejaculatory dysfunction [21]. Although the presence of spermatozoa in post-ejaculatory urine (PEU) should be sufficient for RE diagnosis, there is no consensus on the specific criteria to differentiate true RE from retained ejaculate in the urethra, which is frequently observed in healthy men, and very few studies have been published on this issue [22], [23], [24].

The main objective of this study is to perform a systematic review of the literature available on the different methods currently used to establish a diagnosis of RE. This paper is also intended to provide evidence-based recommendations for the introduction of PEU analysis in clinical practice, and the retrieval of sperm from PEU for use in assisted reproduction techniques (ART).

## Physiology of ejaculation

Ejaculation is a reflex process that involves the emission and expulsion of semen out of the body through the urethra [25]. During emission, sperm is propelled from the epididymis to the prostatic urethra by the contraction of the vas deferens, the seminal vesicles (SV) and the prostate. During this course, the sperm mixes with secretions from the prostate, bulbourethral glands and SV. The prostatic urethra becomes a pressurized compartment where semen accumulates as a result of the contraction of the internal smooth muscle and external striated muscle. Initially, the posterior part of the prostatic urethra and the bladder neck contract, causing the distal portion of the prostatic urethra to dilate. This leads to the accumulation of prostatic and ampullary secretions along with sperm. Then, the SV contract and displace the fluid mixture accumulated in the prostatic urethra, which will constitute the first fraction of ejaculate, and providing the most abundant secretion of the seminal plasma (approximately 70 %), which will constitute the final fraction [1].

During expulsion, the increased pressure in the prostatic urethra, along with the intermittent contraction of the bulbospongiosus and ischiocavernosus muscles that surround the base of the penis, synchronized with the collateral muscles of the pelvic floor, cause the opening of the external sphincter of the prostatic urethra, allowing antegrade ejaculation (AE). During this stage, the neck of the bladder and the internal sphincter of the prostatic urethra remain contracted to prevent ejaculate from flowing back.

Ejaculation, triggered by the mechanical stimulation of the penis, requires the coordination of the sympathetic (which controls the closure of the IUS), parasympathetic, and somatic nervous system (NS). Additionally, the brain plays a modulatory role by inhibiting or stimulating the ejaculatory reflex [1, 2, 26], [27], [28].

### Definition of retrograde ejaculation

RE is a type of post-testicular ejaculatory dysfunction in which, following orgasm, part or all of the semen is propelled into the urinary bladder due to IUS malfunction. RE may be complete, with absence of ejaculate (aspermia), or partial, where an apparently normal ejaculate is observed but with a decrease in seminal volume (hypospermia) and sperm concentration, as well as the presence of sperm in PEU [3, 18, 29].

### Prevalence

RE is a infrequent cause of infertility, accounting for 0.3–2 % of male infertility cases [30]. However, this disorder is involved in 14–18 % of cases of aspermia [31, 32].

### Causes

RE may have an anatomical, pharmacological, endocrine, or neurogenic etiology [2], [3], [4], [5], [6], [7], [8], [9], [10], [11], [12], [13]. Among the most common are the use of drugs (alpha-blockers, antidepressants, antipsychotics, ganglionic blockers), congenital anatomical abnormalities or anatomical changes secondary to surgery that cause IUS incompetence (transurethral prostatectomy, cystectomy, traumatic bladder injury, to name a few), the development of tumors, and radiotherapy treatments in the pelvic area [14]. The most frequent neurogenic causes are neurological disorders involving the loss of sympathetic innervations in the neck of the bladder, including multiple sclerosis, retroperitoneal lymph node dissection without nerve sparing or spinal cord injury, leading to functional bladder neck incompetence [15]. Another common neurogenic cause of RE is secondary neuropathy in patients with poorly controlled DM, causing failure of the sympathetic NS and consequently IUS closure, with a prevalence of 6–34 % depending on different populations and studies [16, 17]. Nevertheless, idiopathic cause appears to be the most common etiology in partial RE (82 %) [15].

### Diagnosis

The presence of aspermia or detection of hypospermia associated with oligozoospermia should raise suspicion of RE [33, 34]. Vroege et al. [35] suggested that the analysis and identification of sperm in PEU support RE diagnosis, although they are not conclusive. In addition, the spectrophotometric detection of fructose in PEU by its colorimetric reaction (yellow–orange) by the indol method helps confirm the presence of RE [18, 36], [37], [38]. It is worth noting that fructose is naturally present in fruits and vegetables and is frequently added to processed foods and drinks, as well as in honey and syrups [39]. In adult males, normal levels of fructose in urine range from undetectable to 0.5298 mmol/L, as measured using highly-sensitive techniques such as high-performance liquid chromatography coupled to tandem mass spectrometry (UPLC-MS/MS: Ultra-performance liquid chromatography-mass spectrometry), even after a high intake of fructose [39, 40]. Fructose concentration in the ejaculate is 25 higher than in urine, with a mean of 14.2 mmol/L in men with proven paternity (MPP) [41] and 13.5 mmol/L in men being evaluated for infertility (MEI) [42]. The presence of fructose concentrations in PEU above baseline levels due to the passage of semen into urine will support diagnosis of RE.

Although diagnosis is established upon confirmation of the presence of sperm in PEU [20], it has been shown that this also occurs in 60–70 % of MPP [22], [23], [24], which casts doubt on the validity of this criterion [43]. Mehta et al. [44] demonstrated that the majority of these spermatozoa are found in the first fraction of urine. Hence, many authors suggest that PEU positivity is most frequently due to the presence of retained semen in the urethra, rather than to true PEU [29]. To prevent false positives in the diagnosis of RE, new diagnostic methods have been developed, such as suprapubic bladder aspiration after orgasm. In this technique, the presence of sperm both in PEU and urine collected from the bladder prior to ejaculation (with higher concentration observed in PEU) would indicate true RE. In contrast, the presence of sperm only in PEU would indicate retained semen in the urethra [34]. Another option is real-time monitoring of ejaculation by transrectal ultrasound, which allows visualization of whether the bladder neck remains open during emission and expulsion [45, 46].

### Treatment

Before initiating any therapy, other potentially reversible etiologies should be excluded, including the use of drugs directly related to the occurrence of RE. Treatment is especially important for patients with RE who wish to have children.

Pharmacotherapy and surgical interventions aimed at treating RE are limited [43]. The literature only includes some small case series and randomized trials. Further studies and randomized placebo-controlled studies are therefore needed [33].

Nevertheless, in the absence of reversible etiologies (anatomical abnormalities, tumors, diabetes), the first-line treatment for RE is pharmacological. This treatment is intended to increase the sympathetic tone both of the IUS and the vas deferens to prevent semen from retrogradely flowing into the bladder. This technique is based on the stimulation of the sympathetic NS or the inhibition of the parasympathetic NS.

– Sympathomimetic drugs are especially useful in patients with slow-progressive disease, such as diabetic neuropathy, or in patients without emission due to an interruption of retroperitoneal sympathetic innervations after surgery. However, many studies available are based on small sample sizes and their results are inconclusive [33]. The most commonly used drugs are synephrine, pseudoephedrine hydrochloride, ephedrine, phenylpropanolamine, and midodrine. These treatments may increase the ejaculatory volume and solve RE in aspermic patients, thereby allowing couples to conceive naturally [47, 48]. Unfortunately, these treatments cause a wide variety of side effects, including dizziness, sleep disturbances, weakness, restlessness, dry mouth, nausea, or sweating – which frequently occur in responders – added to tachycardia and hypertension. Therefore, they should be used with caution in diabetic patients at risk of cardiovascular disease [33, 48], [49], [50], [51].
– The use of parasympatholytic drugs alone, such as brompheniramine maleate and imipramine, has an efficacy of 22 % compared to 39 % when used in combination with sympathomimetics [52]. Hence, combined therapy seems apparently more effective, although statistical analysis is not robust due to small sample sizes [33].
– Other options include buspirone [53] and transurethral injection of collagen or Deflux^®^ (a viscous, biodegradable, biocompatible, and non-migratory gel composed of dextranomer microspheres and non-animal stabilized hyaluronic acid), which can restore the anterograde direction of ejaculation [43].

Finally, although surgical management is successful, only two small case series have been reported in the literature as far back as in the 1980s [52]. Therefore, other options such as the use of sperm retrieved from PEU or from the testes for later use in ART should be considered prior to considering surgery [14].

### Clinical management

Once RE has been confirmed in patients with reproductive desires, and in the case of inefficacy of the proposed treatments, the most frequent, effective, and economical non-surgical alternative in ART is the recovery of viable sperm from PEU [9, 13]. Adequate PEU collection and rapid processing are essential to preserve the viability of sperm.

Three methods for the recovery of sperm from PEU have been described [2, 3, 9, 13, 15, 33, 52].

1. Ejaculation with a full bladder: This technique is intended to achieve AE, as a full bladder can prevent the passage of coagulated ejaculate into the bladder. Masturbation in standing position can contribute to the success of this technique [54]. Even although AE is achieved, there is evidence that part of the ejaculate flows into the bladder. Moreover, AE is not attained in cases of IUS severe incompetence [54]. When AE is not achieved, PEU is examined for sperm [54, 55].
2. Processing of alkalized PEU: The purpose of this technique is to neutralize the acidity of urine to maintain the viability of PEU sperm. Firstly, the urine is alkalinized (by the intake of sodium bicarbonate, acetazolamide, or potassium citrate) or diluted by increasing fluid intake. Then, PEU is obtained by urination or drained via a catheter for later centrifugation [14, 52, 56, 57]. The pellet obtained is resuspended in a culture medium usually supplemented with albumin [5, 57]. The spermatozoa isolated are used for vaginal self-insemination, intrauterine insemination (IUI), conventional *in vitro* fertilization (IVF) or intracytoplasmic sperm injection (ICSI).
3. Hotchkiss method: In this method, the bladder is voided prior to ejaculation using a urethral catheter and then washed and instilled with Ringer’s lactate solution to reduce the acidic ambient of the bladder. Then, the catheter is removed, the patient ejaculates, and the contents of the bladder are emptied by urination or using a new catheter [43, 58]. The modified Hotchkiss technique involves instilling a sterile albumin-containing sperm culture medium into the bladder [59, 60]. The spermatozoa retrieved are used for ART.

Finally, if the retrieval of sperm from PEU fails, a surgical approach can be considered for collecting spermatozoa from the epididymis (PESA: *Percutaneous epididymal sperm aspiration*) or the testes (TESE: *Testicular sperm extraction*) [2]. It is recommended to start with the simplest and least invasive methods, progressively moving to the next method in complexity and invasiveness if unsuccessful.

### PEU processing

#### Pre-analytical phase

##### Patient preparation

Physiological ranges of pH and osmolality values in ejaculated semen are 7.2–8.2 and 300–380 mOsm/kg, respectively [18]. The specific pH and osmolality of urine can damage the plasma membrane, the acrosome and the intermediate piece of spermatozoa, thereby affecting their motility and kinetic parameters [15, 61, 62]. Alterations in osmolality (especially hypoosmolality, as hyperosmolar solutions maintain motility effectively) and the presence of nitrogenous compounds (NH_4_^+^) have more pronounced effects than alterations in pH [5, 31, 33, 59, 63], [64], [65], [66], [67]. Consequently, special attention should be paid to the preparation of RE patients prior to the retrieval of semen and collection of PEU samples (Table 1).

**Table 1:** Instructions for the collection of semen and postejaculation urine for later use of spermatozoa in ART.

2–7 days prior to collection	– Sexual abstinence (3 days recommended)
The day before collection	– Sexual abstinence
	– Drink at least 1.5 L of water throughout the day.
	– Avoid acid foods the evening before: Alcohol, soft drinks, fatty meats, fried foods, vinegar, tomatoes, citrus fruits (lemon, orange, grapefruit, kiwi), bread from refined flours, sugar, yoghurts, cocoa.
	– Have anti-acid foods the evening before: Steamed potatoes, avocado, radishes, pumpkin, broccoli, peas, cucumber, fish and white meats, banana, papaya, milk, oatmeal, chamomile.
	– Take a tablespoon of sodium bicarbonate (2.5 g) diluted in a glass of water (250 mL) 15 min after dinner.
The day of collection	– Upon waking up, urinate to reduce the volume of urine in your bladder as much as possible.
	– Have breakfast as usual around 2 h before going to the laboratory. Avoid drinking coffee, tea, acid foods and tobacco.
	– 15 min after finishing breakfast, take two tablespoons of bicarbonate (approximately 4–5 g) dissolved in two glasses of water (500 mL) 1 h before starting the collection. In diabetic patients, dilute in 3 glasses of water, 750 mL.
	– Go to the laboratory at the scheduled time without having urinated again. There, you will receive two wide-mouth sterile collection containers: * Use one of the containers to try to collect semen by masturbation: The container is labelled as “Semen”. * The other container contains 10 mL of semen washing medium to collect post-ejaculate urine: The container is labelled as “Urine”.
	– Prior to masturbation, urinate again to reduce the volume of urine in your bladder as much as possible^a^.
	– Next, masturbate in standing position and collect the semen, if you ejaculate, in the sterile container labelled as “semen”.
	– After ejaculation, and as soon as possible, collect a sample of urine in the container labelled as “Urine” different from that for semen, containing 10 mL of semen washing medium. Fill only a quarter of the container (until the 25 mL mark).
	– Promptly deliver the two samples to the laboratory for its immediate processing.

^a^In case this urine is collected to measure pH and osmolality, pH should be 7.6–8.1, with osmolality ranging from 300 to 500 mOsm/kg of solution.

##### Collecting semen/PEU samples (following the guidelines of the SEQC^ML^ Working Group on Andrology and Assisted Reproduction Technologies)

Semen samples and analysis are performed according to the 2021 WHO recommendations [18]. If the results of the analysis of semen are suggestive of RE or a sample cannot be obtained (dry orgasm), we recommend that a new attempt be performed on a full bladder in standing position. Then, if AE is achieved, examine the sample of semen. If AE is achieved or results improve, the patient can be suggested either to have sexual intercourse on a full bladder or to collect AE on a full bladder in standing position for later use in ART [54]. If AE is still not obtained, we recommend collecting PEU and analyzing it for the presence of sperm. If PEU contains sperm, a third sample is collected and instructions will be given to previously alkalize urine with oral sodium bicarbonate and increase urine dilution by raising the intake of fluids (Table 1) [68].

The third retrieval will be always performed at the laboratory. First, the patient is instructed to urinate and completely void his bladder. Prior to masturbation, it is necessary to ensure that the pH and osmolality of urine are optimal. If they are suboptimal, the patient will take more bicarbonate. When optimal urine pH and osmolality are achieved, it is necessary to collect AE in a sterile container or at least reach orgasm in cases of aspermia. Then, the patient urinates rapidly 10–50 mL of urine (up to half of the container at most) into another sterile container containing around 10 mL of culture medium for washing spermatozoa preheated to 37 °C and supplemented with human serum albumin (it helps maintain pH and osmolality of semen in urine). Finally, the patient will deliver both samples to the laboratory staff within a maximum period of 5 min after collection [15, 69].

#### Analytical phase

For the correct evaluation of RE, the patient is required to provide a sample of PEU and semen, in case he achieved AE [15, 70].

##### Technical considerations in relation to PEU analysis by microscopy

For the examination of urine samples, 10x magnification objectives are considered to be low-power (LPF, low power microscopy field) whereas 20× and 40× objectives are considered high-power (HPF, high power microscopy field). The recent UNE-EN ISO 23162:2022 standard recommends the use of both, 20× and 40× for examining samples for sperm (Section 6.3.6). However, the standard only defines 40× as HPF [71]. On the other hand, the volume of sample observed in each microscopic field depends on the field area (circle) and the depth of the preparation/counting chamber (i.e. 20.7 µm for a fresh preparation of 10 µL on a slide and covered with a coverslip of 22 × 22 mm, #1.5 or #2 thickness). The radius of the microscopic field can be measured using an eyepiece micrometer or can be estimated by dividing the diameter of the ocular aperture by the magnification of the objective lens.

For example, with a HPF 40× objective and a 10× ocular with an aperture of 20 mm: Four spermatozoa per field of view correspond to 1 × 10^6^ spermatozoa/mL, and with a HPF 20x objective and an 10 × ocular with an aperture of 20 mm: 16 spermatozoa per field of view correspond to 1 × 10^6^ spermatozoa/mL [18]. With a 10x ocular with a 22 mm aperture, the equivalent would be 5 and 20 spermatozoa per field of view, respectively (Table 2).

**Table 2:** Equivalent in millions/mL of observed spermatozoa/field of view as a function of the ocular and objective, observed at a depth of field of the preparation of 20.7 µm (10 µL of sample placed on the slide and coverslip of 22 × 22 mm^a^).

10× ocular + 20× objective						
	Field diameter^b^	Radius of field (r)	r^2^	Area of field (A)^c^	Field volume^d^	Number of spermatozoa/field^e^
20 mm ocular aperture	1 mm	500 µm	250,000 µm^2^	785,500 µm^2^	16,259,850 µm^3^≈16 × 10^−6^/mL	≈16
22 mm ocular aperture	1.1 mm	550 µm	302,500 µm^2^	950,332 µm^2^	19,671,872 µm^3^≈20 × 10^−6^/mL	≈20

10× ocular + 40x objective						
	Field diameter^b^	Radius of field (r)	r^2^	Area of field (A)^c^	Field volume^d^	Number of spermatozoa/field^e^
20 mm ocular aperture	0.5 mm	250 µm	62,500 µm^2^	196,375 µm^2^	4,064,962 µm^3^≈4 × 10^−6^/mL	≈4
22 mm ocular aperture	0.55 mm	275 µm	75,625 µm^2^	237,583 µm^2^	4,917,968 µm^3^≈5 × 10^−6^/mL	≈5

^a^Coverslip with thickness #1.5 – 0.16 to 0.19 mm – or #2 – 0.19 to 0.23 mm –. ^b^Diameter of the field=ocular aperture in mm/objective magnification. ^c^Area of field (A=πr^2^). ^d^Volume of field=A × depth of field. ^e^Number of spermatozoa/field equivalent to 1 × 10^6^/mL.

To determine the presence of sperm in PEU, we recommend:

– Use a 10 × ocular lens (22-mm-aperture).
– Dispense a 10 uL aliquot between a slide and a coverslip of 22 × 22 mm (thickness #1.5 – 0.16 to 0.19 mm – or #2 – 0.19 to 0.23 mm –), which will ensure the complete spread of the sample.

##### Processing and analysis of ejaculate and PEU specimens for ART (according to recommendations of the SEQC^ML^ Working Group on Andrology and Assisted Reproduction Technologies)

IF AE is achieved, the sample is analyzed following the 2021 WHO guidelines [18].

**Figure 1:**
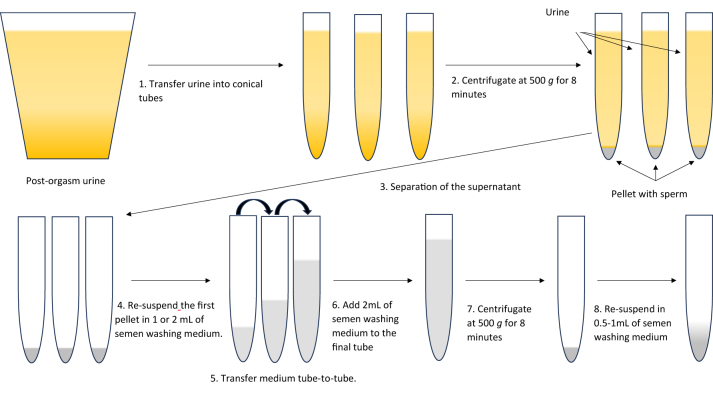
Retrieval of sperm from a PEU sample.

Then, the PEU is collected and an aliquot is examined to determine its pH and osmolality. Next, the sample is examined for the presence of sperm [15, 70, 72]. It is worth noting that spermatozoa in PEU are extremely fragile, as urine can damage the intermediate piece [61]. Therefore, the sample must be handled with extreme care [57]. As soon as the laboratory receives the sample, it is loaded onto a counting chamber and an aliquot of 10 µL is placed on a slide and a coverslip [71]. Then, the PEU is split into aliquots of 10 mL and stored in sterile conical tubes. The total urine volume is recorded and the tubes are centrifuged at 500 *g* for 8 min [18] at room temperature to rapidly separate sperm from urine. During centrifugation, the counting chamber is manually or automatically examined. If the presence of sperm is confirmed, their concentration and motility is calculated. Otherwise, if sperm is not detected by the counting chamber, the 484 fields of the slide are evaluated. If sperm is identified, their count and motility observed in each field are noted down. Results will be expressed as means, calculated by dividing the total sperm count by the total of observed fields [15, 70, 72]. After urine tube centrifugation, the supernatant is separated without disturbing the precipitate. Next, the pellet of the first tube is re-suspended in 2 mL of sperm washing medium, preheated at 37 °C, and the resulting solution is pipetted into the second tube to re-suspend its pellet. This procedure is repeated sequentially until all pellets have been re-suspended. Another 2 mL of medium are added to the resulting solution, which is centrifuged at 500 *g* for 8 min at room temperature. After centrifugation, the supernatant is separated without disturbing the pellet. Then, the pellet is re-suspended in 0.5–1 mL of medium (as a function of the initial concentration and observed pellet) and the total volume obtained is recorded (Figure 1). Finally, sperm concentration and motility are manually or automatically evaluated, as with the precentrifugation PEU sample. The sperm recovered can be used directly for ART or cryopreserved for later use [52, 72], [73], [74].

#### Post-analytical phase

##### Criteria for a sperm count in PEU to be considered positive

Although the mere presence of sperm in PEU should be conclusive for a diagnosis of RE, cut-offs for PEU sperm have not yet been established and the literature on this subject is very limited [4]. Three criteria have been proposed to date:

1. Arbitrarily selecting an absolute sperm count in PEU above which the test will be considered positive: Although clear cut-offs have not been established, a result of 10–15 spermatozoa/HPF after centrifugation at 300 *g* for 10 min [2, 15, 21, 22, 44, 75], or the presence of more than one million spermatozoa [76], are considered diagnostic criteria for RE. It is worth mentioning that the first initial studies applying this criterion used microscopes with 20 mm oculars lenses, whereas modern oculars are 22 mm.
  Consequently, following WHO recommendations [18], the SEQC^ML^ Working Group on Andrology and Assisted Reproduction Technologies recommend using a cut-off of 12–20 sperm/20x HPF (0.625–0.94 millions/mL), which corresponds to the equivalent with 22 mm ocular lenses, after 8 min centrifugation at 500 *g* of PEU.
2. Arbitrarily selecting a cut-off for the percentage sperm count in PEU relative to the index of retroejaculation (IR). The IR is calculated by dividing the total sperm count in PEU by the total sperm count in the ejaculate (which corresponds to the sum of the total count in PEU plus the total count in the ejaculate); then, the result is multiplied by 100. This option is only applicable if an ejaculate is available (the less frequent form of RE). Some authors propose this option because they are hesitant to consider the mere presence of sperm in PEU as confirmatory of an RE diagnosis [77].
  The SEQC^ML^ Working Group on Andrology and Assisted Reproduction Technologies adopt the 97.5th percentile proposed by Ariagno et al. [77]. Hence, MEI with a total sperm count in PEU>3.8 × 10^6^ and with an IR>2.16 % are considered retroejaculators [77].
3. However, these criteria cannot be applied when the patient is azoospermic and has concomitant RE, since sperm cannot be identified in PEU. In this setting, the detection of fructose in a pre-masturbation urine sample (negative control) and in PEU (test) can aid diagnosis, provided that fructose levels in PEU exceeds baseline values [5, 18, 33, 37, 38, 78]. This criterion will not be applicable to patients with azoospermia and SV agenesis, as fructose cannot be found in PEU.
  In contrast, this criterion can serve as a third diagnostic criterion for RE in the presence of AE. Fructose detection will be performed in a pre-masturbation urine sample (negative control), in AE (positive control) and PEU (test).

The SEQC^ML^ Working Group on Andrology and Assisted Reproduction Technologies suggest fructose values in PEU>0.53 mmol/L as confirmatory of RE, whether it is determined by the indol, enzymatic or chromatographic method.

##### Practical difficulties in applying criteria to consider a positive OPE result

The application of any of the three proposed criteria in clinical practice is complex. Firstly, the presence of sperm in PEU may be due either to a true RE in the bladder or to the residual ejaculate that remains in the urethra and is later washed-out during voiding, an event with intra and inter-subject variability.

In men with AE, most of the sperm found in the urethra following ejaculation are observed in the first fraction of the urine (generally 10–20 mL) and expelled during the first void [23]. In addition, the probability to detect sperm increases as the sperm count in the ejaculate increases and the time elapsed since ejaculation shortens [23, 44, 79]. Hence, there is evidence that spermatozoa can be observed in most of the PEU samples collected 0.5–4 h after ejaculation (in 59.5 % of PEU samples at 30 min and in 70 % at 2 h, with them becoming undetectable at 5 h). Motile spermatozoa can still be found in the PEU up to 4.5 h after ejaculation [23]. Hence, excluding cases of aspermia, when the sperm count in the AE is <5 million/mL and seminal volume <0.5 mL, PEU should be examined for sperm instead of directly indicating ICSI. The reason is that RE could be treatable or an adequate number of sperm may be recovered in the urine to pursue an IUI [44]. In any case, these findings indicate that the most common reason for a positive PEU is the wash-out of semen retained in the urethra, rather than a true RE.

##### ART available with sperm retrieved from PEU

The recovery of sperm in MEI with complete RE is unpredictable and highly variable. For this reason, cryopreservation of sperm from PEU prior to the ART is recommended [60].

Regarding the processing of sperm from PEU, available options range from simple washing and centrifugation [54, 57, 80] to more advanced selection techniques such as density gradient centrifugation [74, 80, 81] or swim-up methods [82], [83], [84]. Pregnancy can be naturally achieved after a previous failed ICSI by means of a transurethral injection of Deflux^®^ in the neck of the bladder, which restores AE [43].

## Conclusions

PEU screening should be performed in MEI cases including aspermia, hypospermia with associated azoospermia, hypospermia with associated oligozoospermia, and in patients with a history of urological surgery or distal urethral stenosis.

This is the first work to describe arbitrary criteria for RE adapted to 22 mm oculars. Hence, the presence of >12–20 spermatozoa/HPF with a 20× objective and a 22 mm ocular lenses following PEU centrifugation could be suggestive of RE. Likewise, the presence of fructose in PEU is an additional parameter supporting diagnosis.

Due to its simplicity, effectiveness and non-invasive nature, the oral intake of a urinary alkalinizing agent such as sodium bicarbonate is recommended to regulate urinary pH and osmolality, thus optimizing the PEU sample and the isolation of sperm for later use in ARTs. Additionally, there are several types of waste products that are cleared in urine that may affect sperm. However, their levels can be minimized by inducing diuresis before attempting ejaculation and PEU collection.

Compliance with the special conditions described for collecting PEU is essential. Prompt post-ejaculation voiding is required to increase the probability of finding the greatest number of live and motile sperm in the PEU sample.

In couples in whom pharmacological treatment for RE was unsuccessful, the use of ARTs based on the retrieval of sperm from a PEU sample or directly from the epididymis or the testes emerges as the only option for achieving pregnancy with the couple’s own gametes.
